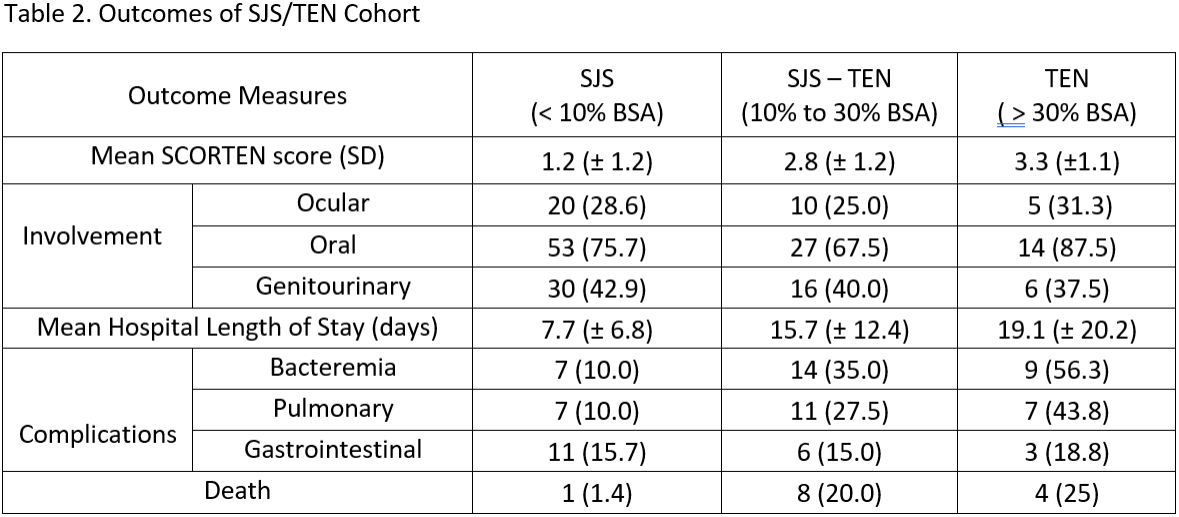# 294 Outcomes in Stevens-johnson Syndrome/toxic Epidermal Necroylsis Patients Treated Without Admission to a Verified Burn Center

**DOI:** 10.1093/jbcr/irad045.269

**Published:** 2023-08-29

**Authors:** Joseph Lewcun, Michael J Feldman, Christina Kontzias, Megan Newsom, Alex Vagonis

**Affiliations:** VCU Health System, Richmond, Virginia; VCU Health System, Richmond, Virginia; VCU Health System, Richmond, Virginia; VCU Health System, Richmond, Virginia; VCU Health System, Richmond, Virginia

## Abstract

**Introduction:**

The care of patients with Stevens-Johnson Syndrome (SJS) and Toxic Epidermal Necroylsis (TEN) has traditionally been delivered within a burn center. Our burn center utilizes a multi-disciplinary approach which involves admission to the medicine service, with support from the burn team. The aim of this study is to determine whether SJS/TEN patients cared for with our system, including burn involvement but not burn admission, demonstrate equivalent outcomes.

**Methods:**

A retrospective review of all SJS/TEN patients admitted to the medicine service at a single academic medical center from 2009 to 2021 was conducted. Demographic characteristics and outcome measures such as mortality, length of intensive care unit (ICU) stay and total length of hospitalization were collected. The Severity-of-Illness Score for Toxic Epidermal Necrolysis (SCORTEN) was used to calculate expected mortality rates within the cohort [2]. The observed mortality rates were then compared to the expected mortality rates.

**Results:**

126 patients who were admitted for SJS/TEN were included (70 SJS, 40 SJS/TEN overlap, 16 TEN). The mortality rate for the entire cohort was 10.32% as compared to a 22.33% expected mortality rate (p=0.010). The observed and expected mortality rates for SJS, SJS/TEN overlap, and TEN sub-groups were 1.43% observed versus 10.22% expected (p=0.029), 20.00% observed versus 35.83% expected (p=0.133), and 25.00% observed version 44.06% expected (p=0.264) respectively. The mean ICU length of stay was 3.58 days, and the mean length of total hospitalization was 11.69 days.

**Conclusions:**

Mortality rates in SJS/TEN patients admitted to medicine units are equivalent or decreased as compared to SCORTEN predicted mortality rates.

**Applicability of Research to Practice:**

Admission of SJS/TEN patients to a medicine unit is appropriate as long as there is burn team involvement in their care, which may be more suitable for patients who require management of complex medical comorbidities.